# Supplementary material for: Modifications at K31 on the lateral surface of histone H4 contribute to genome structure and expression in apicomplexan parasites
Source: eLife. 2017 Nov 4;6:e29391. doi: 10.7554/eLife.29391 (PMC5685513; doi:10.7554/eLife.29391)
Supplement: Transparent reporting form [file elife-29391-transrepform.pdf]

1st Floor
24 Hills Road
Cambridge CB2 1JP, UK

P 01223 855340
W elifesciences.org
T @elife

# *eLife's* transparent reporting form

We encourage authors to provide detailed information *within their submission* to facilitate the interpretation and replication of experiments. If you have any questions, please contact us: editorial@elifesciences.org.

## Sample-size estimation

- You should state whether an appropriate sample size was computed when the study was being designed
- You should state the statistical method of sample size computation and any required assumptions
- If no explicit power analysis was used, you should describe how you decided what sample (replicate) size (number) to use

Please outline where this information can be found within the submission (e.g., page numbers or figure legends), or explain why this information doesn't apply to your submission:

Sample size estimation was not performed.
All the experiments were performed at least in triplicate independent experiments in accordance with well-established reporting procedures for all the assays.
For the mass spectrometry analysis, 3 sets of biological replicates (semi-purified nucleosome) were analyzed but only one experiment was able to identify with high confidence H4K31ac.

## Replicates

- You should report how often each experiment was performed
- You should include a definition of biological versus technical replication
- The data obtained should be provided and sufficient information should be provided to indicate the number of independent biological and/or technical replicates
- If you encountered any outliers, you should describe how these were handled
- Criteria for exclusion/inclusion of data should be clearly stated
- High-throughput sequence data should be uploaded before submission, with a private link for reviewers provided (these are available from both GEO and ArrayExpress)

Please outline where this information can be found within the submission (e.g., page numbers or figure legends), or explain why this information doesn't apply to your submission.

Immunofluorescence and cas9-inactivation data are representative of three to four independent experiments as indicated in figure legends.

eLife Sciences Publications, Ltd is a limited liability non-profit non-stock corporation incorporated in the State of Delaware, USA, with company number 5030732, and is registered in the UK with company number FC030576 and branch number BR015634 at the address 1st Floor, 24 Hills Road, Cambridge CB2 1JP | August 2014

The source data and processed files from the *Toxoplasma gondii* ChIP-seq datasets has been deposited via GEO DataSets under the accession number : Series GSE98806. *Plasmodium falciparum* ChIPseq raw and processed files can be found at NCBI Bioproject ID PRJNA386433

**Statistical reporting**

- Statistical analysis methods should be described and justified
- Raw data should be presented in figures whenever informative to do so (typically when N per group is less than 10)
- For each experiment, you should identify the statistical tests used, exact values of N, definitions of center, methods of multiple test correction, and dispersion and precision measures (e.g., mean, median, SD, SEM, confidence intervals; and, for the major substantive results, a measure of effect size (e.g., Pearson's r, Cohen's d)
- Report exact p-values wherever possible alongside the summary statistics and 95% confidence intervals. These should be reported for all key questions and not only when the p-value is less than 0.05.

Please outline where this information can be found within the submission (e.g., page numbers or figure legends), or explain why this information doesn't apply to your submission:

As shown in Fig. 8_supplement figure S1A and B, S3A and Fig. 10 supplement figure 1 - ChIP-seq analysis : Correlation of the different biological replicates were calculated by performing Pearson's and Spearman's correlation analysis of pairwise comparison of BAM alignment files, and ChIP-seq peak enrichment scores (log2) using MACS2 and deepTools.

As indicated in M&M (lines 834-840 page 26) for Peak detection: The mapped reads were used for peak detection by MACS v1.4.0 (Model-based Analysis of ChIP-Seq) software. Statistically significant ChIP-enriched regions (peaks) were identified by comparison to a Poisson background model (Cut-off p-value=10e-5).

**Solexa CHASTITY Quality Filter:** Individual bases generated from original image files have quality scores which reflect the probability whether a base-call is correct or not. The score is quantified by CHASTITY Formula (as shown in the figure right).
The CHASTITY(C) of each base in the short reads is determined by the intensity of four colors ($I_A$, $I_C$, $I_G$, $I_T$ here), and the formula means "the ratio of the highest ($I_C$ here) of the four (base type) intensities to the sum of highest two ($I_C$ and $I_G$ here)." The CHASTITY(C) should be no less than 0.6 in the first 25 bases

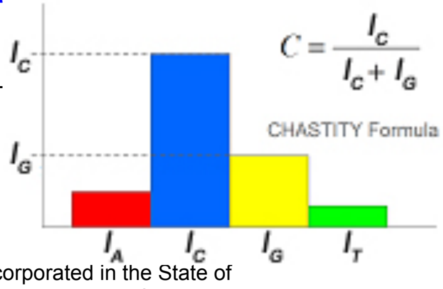

(For large datasets, or papers with a very large number of statistical tests, you may upload a single table file with tests, Ns, etc., with reference to page numbers in the manuscript.)

**Additional data files ("source data")**

- We encourage you to upload relevant additional data files, such as numerical data that are represented as a graph in a figure, or as a summary table
- Where provided, these should be in the most useful format, and they can be uploaded as "Source data" files linked to a main figure or table
- Include model definition files including the full list of parameters used
- Include code used for data analysis (e.g., R, MatLab)
- Avoid stating that data files are "available upon request"

Please indicate the figures or tables for which source data files have been provided:

Figure 9D_source data_1.xlsx: Figure 9 (Panel D) : RNA-Seq of Pru in BMDM

The source data and processed files from the *Toxoplasma gondii* ChIP-seq datasets has been deposited via GEO DataSets under the accession number : Series GSE98806.

https://www.ncbi.nlm.nih.gov/geo/query/acc.cgi?acc=GSE98806

At this web site raw data are available but also Bed and WIG processed data:
- the EXCEL/BED format file containing the ChIP-enriched regions was generated for each sample. We use 10-bp resolution intervals (10-bp bins) to partition the stacked reads region, and count the number of reads in each bin.
- All the 10 bp resolution ChIP-seq profiles of each sample are saved as UCSC wig format files, which can be visualized in Toxoplasma gondii Genome Browser.

*Plasmodium falciparum* ChIPseq raw and processed files can be found at NCBI Bioproject ID PRJNA386433
